# Upfront Chemotherapy Followed by Stereotactic Body Radiation Therapy with or without Surgery in Older Patients with Localized Pancreatic Cancer: A Single Institution Experience and Review of the Literature

**DOI:** 10.3390/curroncol29010028

**Published:** 2022-01-11

**Authors:** Abhinav V. Reddy, Shuchi Sehgal, Colin S. Hill, Lei Zheng, Jin He, Joseph M. Herman, Jeffrey Meyer, Amol K. Narang

**Affiliations:** 1 Department of Radiation Oncology & Molecular Radiation Sciences, Sidney Kimmel Cancer Center, Johns Hopkins University School of Medicine, 401 N Broadway, Baltimore, MD 21231, USA; ssehgal3@jhu.edu (S.S.); chill48@jhmi.edu (C.S.H.); jmeyer58@jhmi.edu (J.M.); anarang2@jhmi.edu (A.K.N.); 2 Department of Oncology, Sidney Kimmel Cancer Center, Johns Hopkins University School of Medicine, 401 N Broadway, Baltimore, MD 21231, USA; Lzheng6@jhmi.edu; 3 Department of Surgery, Sidney Kimmel Cancer Center, Johns Hopkins University School of Medicine, 401 N Broadway, Baltimore, MD 21231, USA; jhe11@jhmi.edu; 4 Department of Radiation Oncology, Northwell Health, 450 Lakeville Road, New Hyde Park, NY 11042, USA; jherman1@northwell.edu

**Keywords:** stereotactic body radiation therapy, SBRT, older patients, pancreatic cancer, localized pancreatic cancer, clinical outcomes, toxicity

## Abstract

Objective: To report on clinical outcomes and toxicity in older (age ≥ 70 years) patients with localized pancreatic cancer treated with upfront chemotherapy followed by stereotactic body radiation therapy (SBRT) with or without surgery. Methods: Endpoints included overall survival (OS), local progression-free survival (LPFS), distant metastasis-free survival (DMFS), progression-free survival (PFS), and toxicity. Results: A total of 57 older patients were included in the study. Median OS was 19.6 months, with six-month, one-year, and two-year OS rates of 83.4, 66.5, and 42.4%. On MVA, resection status (HR: 0.30, 95% CI 0.12–0.91, *p* = 0.031) was associated with OS. Patients with surgically resected tumors had improved median OS (29.1 vs. 7.0 months, *p* < 0.001). On MVA, resection status (HR: 0.40, 95% CI 0.17–0.93, *p* = 0.034) was also associated with PFS. Patients with surgically resected tumors had improved median PFS (12.9 vs. 1.6 months, *p* < 0.001). There were 3/57 cases (5.3%) of late grade 3 radiation toxicity and 2/38 cases (5.3%) of Clavien-Dindo grade 3b toxicity in those who underwent resection. Conclusion: Multimodality therapy involving SBRT is safe and feasible in older patients with localized pancreatic cancer. Surgical resection was associated with improved clinical outcomes. As such, older patients who complete chemotherapy should not be excluded from aggressive local therapy when possible.

## 1. Introduction

Pancreatic cancer is the third leading cause of cancer-related deaths in the United States, responsible for over 48,000 deaths each year [1]. In fact, it is projected to become the second most common cause of cancer-related deaths by the year 2040 [2]. Treatment of localized disease usually involves a combination of chemotherapy, radiation therapy, and surgical resection [3]. Unfortunately, even with aggressive therapy, outcomes are guarded with a five-year overall survival (OS) rate of less than 15% for borderline resectable and locally advanced pancreatic cancer (BRPC/LAPC) [4].

Pancreatic cancer can be considered a disease of older patients with the median age of diagnosis of 70 years [5]. In fact, two thirds of newly diagnosed patients are over 65 years [5]. As our population continues to age, the incidence of pancreatic cancer in the older patients is expected to increase [6]. However, optimal management of this group of patients is largely unknown given that clinical trials consist predominantly of younger patients, and as such, the results cannot necessarily be applied to the treatment of older patients [7,8]. Furthermore, only a small proportion of older patients currently receive what would be considered standard of care therapy for non-older patients [9,10,11,12]. In fact, one study demonstrated that only 44% of patients over 65 years with LAPC received any treatment at all [13]. As such, more information is needed regarding the optimal management of older patients with pancreatic cancer.

Older patients are more likely to have co-morbidities or poor performance status that precludes aggressive therapy such as surgical resection and adjuvant therapy [9]. Stereotactic body radiation therapy (SBRT), which is used for the purpose of margin sterilization in the neoadjuvant setting or for improved local progression-free survival (LPFS) in the definitive/unresectable setting, may have a role in these patients. There have been only a handful of studies investigating the role of SBRT in older patients [14,15,16,17,18]. Unfortunately, many of these studies are limited by heterogeneous treatment and disease characteristics. As such, we report on a cohort of older patients (age ≥ 70 years) with BRPC/LAPC who were treated with upfront chemotherapy followed by five-fraction SBRT with or without surgery. We report on clinical outcomes such as OS, LPFS, distant metastasis-free survival (DMFS), and progression-free survival (PFS), as well as on toxicity. We also compare practice patterns between older patients and patients < 70 years.

## 2. Materials and Methods

### 2.1. Study Design

This was a single institution retrospective review of patients with BRPC/LAPC who were treated with upfront chemotherapy followed by SBRT with or without surgery, with a focus on older patients (≥70 years), which comprised the study population. The study was approved by our institutional review board. The inclusion criteria were as follows: (1) Biopsy proven diagnosis of pancreatic cancer; (2) Age ≥ 70 years; (3) BRPC or LAPC per NCCN criteria [3]; (4) Treatment with upfront chemotherapy followed by SBRT; (5) Regular follow-up with diagnostic imaging available for review. Clinical outcomes and toxicity were evaluated for older patients. In addition, practice patterns of older patients were compared to that of the rest of the source population (<70 years). Radiation and chemotherapy-related toxicity were evaluated with the Common Terminology Criteria for Adverse Events (CTCAE) criteria, and postoperative toxicity was assessed within 90 days after surgery with Clavien–Dindo classification.

### 2.2. Definition of Clinical Outcomes

Clinical outcomes included OS, LPFS, DMFS, and PFS. Overall survival was defined as time from completion of SBRT to death. Patients who were alive were censored at time of last clinic visit. Local-progression free survival and DMFS were defined as time from completion of SBRT to development of locoregional disease and distant disease on imaging, respectively. Death was not included as an endpoint when evaluating LPFS and DMFS. Patients who did not experience local progression or distant disease were censored at time of last imaging follow-up. Progression-free survival was defined as time from completion of SBRT to development of any radiographic evidence of disease progression or death. Patients who did not experience any progression were censored at time of last imaging follow-up.

### 2.3. General Treatment Paradigm

At our institution, all patients with BRPC/LAPC were treated with upfront chemotherapy, with a preference for multi-agent regimens when feasible. During chemotherapy, pancreatic protocol computed tomography (CT) scans were performed at approximately three-month intervals to assess treatment response. After completion of chemotherapy, patients were recommended for five-fraction SBRT if they had stable or responding disease. After completion of SBRT, patients had re-staging diagnostic imaging. All BRPC patients were taken for surgical exploration if they had imaging evidence of disease response/stability and no medical contraindications. The same was true for LAPC, with the exception of patients with extensive local disease characterized by encasement of multiple arterial structures or occlusion of venous structures with collateral formation, precluding a reasonable pathway for surgical resection.

### 2.4. SBRT Treatment Details

After completion of upfront chemotherapy, patients were planned for five-fraction SBRT. Prior to simulation, all patients had endoscopic ultrasound-guided placement of gold fiducials to assist with image guidance. At time of simulation, patients were placed supine with arms above head and immobilized in a Vac-Lok (CIVCO Medical Solutions, Coralville, IA, USA). Thin-sliced CT scans with intravenous contrast were acquired for treatment planning. Target volumes and organs at risk were delineated using Pinnacle Treatment Planning System (Phillips Radiation Oncology Systems, Fitchburg, WI, USA). Active breathing control (ABC, Elekta, Stockholm, Sweden) was utilized for motion management. Patients who could not tolerate breath hold underwent a four-dimensional CT scan, and an internal target volume (ITV) was generated from the maximum inspiratory and expiratory phases. The clinical target volume (CTV) consisted of gross disease on imaging in addition to full circumference of involved vasculature. The planning target volume (PTV) was generated by adding 3–5 mm isotropic expansion to the CTV in breath-hold cases and to the ITV in free breathing cases. Daily image guidance with pre-treatment and intrafraction cone beam CT scans was performed to ensure appropriate patient positioning. Patients were aligned to bone and then shifted to align to fiducials. All patients were treated on an Elekta linear accelerator unit (Elekta, Stockholm, Sweden).

### 2.5. Statistical Analysis

Patient, treatment, and disease characteristics were recorded including age, sex, Eastern Cooperative Oncology Group score, Adult Comorbidity Evaluation-27 score, tumor location, disease extent, baseline cancer antigen 19-9 (CA 19-9) and total bilirubin, chemotherapy type and duration, SBRT dose and fractionation, PTV size, and resection status. Univariate Cox analysis (UVA) was performed to evaluate associations between the aforementioned variables with clinical outcomes. Variables with *p* < 0.05 on UVA were entered into multivariable Cox analysis (MVA). Kaplan-Meier analysis was performed for survival outcomes, and log-rank test was used to assess significance between groups. Chi-square and Mann–Whitney U tests were used to assess differences in practice patterns and toxicity between various groups. A *p* value < 0.05 was considered significant and all *p* values were two-sided. Statistical analysis was performed with JMP version 15.0 (SAS institute, Cary, NC, USA)

## 3. Results

### 3.1. Patient, Disease, and Treatment Characteristics

Patient, disease and treatment details are displayed in Table 1. From August 2016 to May 2021, a total of 182 patients were treated with upfront chemotherapy and SBRT, of which 57 patients had age ≥ 70 years and comprised the study population. The median age was 73.6 years (range, 70.1–84.1 years). There were 31 males (54.4%) and 26 females (45.6%). Eastern cooperative group score of 0, 1, and 2 were seen in 17 (29.8%), 38 (66.7%), and 2 (3.5%) patients, respectively. Adult Comorbidity Evaluation-27 score was 0, 1, 2, and 3 in 11 (19.3%), 35 (61.4%), 9 (15.8%), and 2 (3.5%) patients, respectively. Disease extent was borderline resectable in 27 patients (46.4%) and locally advanced in 30 patients (52.6%). Baseline CA 19-9 and total bilirubin were 233.3 U/mL (range, 1.0–7358.4 U/mL) and 0.55 U/mL (range, 0.2–15.2 U/mL), respectively. Median induction chemotherapy duration was four months (range, 2–18 months) and consisted of modified FOLFIRINOX (mFFX) (30/57, 52.5%), gemcitabine plus nab-paclitaxel (GnP) (24/57, 42.1%), mFFX and capecitabine (1/57, 1.8%), gemcitabine and capecitabine (1/57, 1.8%), and gemcitabine (1/57, 1.8%). The most common SBRT dose fractionation was 33 Gy in five fractions (53/57, 93.0%), followed by 30 Gy in five fractions (2/57, 3.5%), and 36 Gy in five fractions (2/57, 3.5%). Median PTV was 108.8 cc (range, 13.1–368.9 cc). The majority of patients were treated with breath hold technique (43/57, 75.4%). The majority of patients underwent surgical resection (38/57, 66.7%), with Whipple procedure (27/38, 71.1%), distal pancreatectomy (10/38, 26.3%), or total pancreatectomy (1/38, 2.6%). Of the 19 patients who did not undergo surgical resection, two had medical contraindications/advanced age, six had extensive local disease characterized by encasement of arterial structures or occlusion of venous structures with collateral formation, six developed distant disease on re-staging CT prior to surgery, and five had evidence of distant disease at time of surgical exploration. Post-SBRT/surgery chemotherapy was administered to 19 patients (33.3%) for a median duration of two months (range, one–six months).

### 3.2. Practice Patterns

Table 2 shows practice patterns in the study population (≥70 years) compared to the rest of the source population (<70 years). Multi-agent chemotherapy was administered to a similar proportion in both populations (98.2% vs. 100, *p* = 0.127). Older patients were more likely to receive induction GnP (42.1% vs. 12.2%, *p* < 0.001). Median duration of chemotherapy was similar between both groups (*p* = 0.233). Older patients were less likely to undergo surgical exploration (75.4% vs. 88.8%, *p* = 0.025). Post-operative/SBRT chemotherapy was administered to a similar proportion in both groups (33.3% vs. 36.0%, *p* = 0.726).

Supplementary Table S1 shows practice patterns and treatment related toxicity in the study population based on three age cutoffs: 70–75, 75–80, and > 80 years. Management was similar in all three groups with the exception of chemotherapy regimen, with GnP given more frequently to older patients (32.4% vs. 42.9% vs. 100%, *p* < 0.003). There was no difference in treatment related toxicity between the three groups.

### 3.3. Clinical Outcomes

The median follow-up after SBRT for the entire cohort was 13.5 months (range, 0.63–50.4 months). At time of last follow-up, 27 patients (47.4%) were alive. Patterns of failure at last follow-up were as follows: 3 with local failure alone, 20 with distant failure alone, and 18 with local and distant failure. Of the 18 patients with local and distant disease, 3 developed local failure first, 3 developed distant failure first, and 12 developed synchronous local and distant failure.

**Figure 1:**
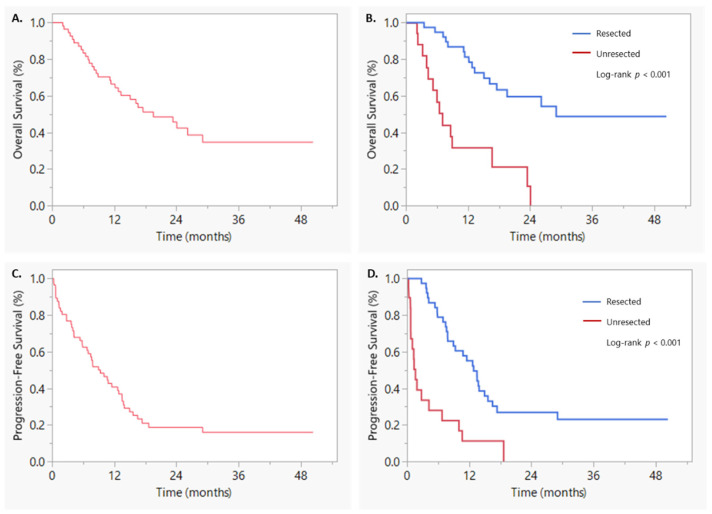
Kaplan–Meier curves of (**A**) overall survival, (**B**) overall survival based on resection status, (**C**) progression-free survival, and (**D**) progression-free survival based on resection status.

Median OS was 19.6 months, with six-month, one-year, and year-year OS rates of 83.4, 66.5, and 42.4%, respectively (Figure 1A). On UVA, sex (HR: 2.22, 95% CI 1.03–4.76, *p* = 0.042), ACE-27 (HR: 0.35, 95% CI 0.16–0.74, *p* = 0.007), resection status (HR: 0.20, 95% CI 0.092–0.42, *p* < 0.001), and post-SBRT/surgery chemotherapy (HR: 0.26, 95% CI 0.11–0.64, *p* = 0.004) were associated with OS. Of note, age was not associated with OS on UVA. On MVA, only resection status (HR: 0.30, 95% CI 0.12–0.91, *p* = 0.031) was associated with OS (Table 3). Patients with surgically resected tumors had a median OS of 29.1 versus 7.0 months in patients with unresected tumors (log-rank *p* < 0.001) (Figure 1B). Median LPFS was 18.7 months with six-month, one-year, and two-year LPFS rates of 86.8, 73.3, and 44.6% (Supplementary Figure S1A). On UVA, only pre-SBRT CA 19-9 U/mL was associated with LPFS (HR: 1.002, 95% CI 1.00–1.005, *p* = 0.037) (Supplementary Table S2). Of note, resection status was not associated with LPFS (Supplementary Figure S1B). As such, MVA was not performed for LPFS.

Median DMFS was 13.5 months, with six-month, one-year, and two-year DMFS rates of 67.2, 53.0, and 31.6%, respectively (Supplementary Figure S1C). On UVA, resection status (HR: 0.22, 95% CI 0.11–0.46, *p* < 0.001), ACE-27 (HR: 0.34, 95% CI 0.16–0.72, *p* = 0.005) and post-SBRT/surgery chemotherapy (HR: 0.47, 95% CI 0.23–0.94, *p* = 0.032) were associated with DMFS. On MVA, only resection status (HR: 0.33, 95% CI 0.14–0.77, *p* = 0.010) was associated with DMFS (Supplementary Table S3). Patients with surgically resected tumors had a DMFS of 15.6 versus 1.6 months in patients with unresected tumors (log-rank, *p* < 0.001) (Supplementary Figure S1D). Median PFS was 9.3 months, with six-month, one-year, and two-year PFS rates of 62.5, 40.7, and 18.6%, respectively (Figure 1C). On UVA, age (HR: 1.09, 95% CI 1.01–1.18, *p* = 0.034), sex (HR: 1.89, 95% CI 1.04–3.46, *p* = 0.038), ACE-27 (HR: 0.33, 95% CI 0.16–0.66, *p* = 0.002), resection status (HR: 0.25, 95% CI 0.13–0.47, *p* < 0.001), and post-SBRT/surgery chemotherapy (HR: 0.43, 95% CI 0.22–0.82, *p* = 0.011) were associated with PFS. On MVA, only resection status (HR: 0.40, 95% CI 0.17–0.93, *p* = 0.034) was associated with PFS (Table 4). Patients with surgically resected tumors had a median PFS of 12.9 versus 1.6 months in unresected patients (log-rank *p* value < 0.001) (Figure 1D).

### 3.4. Toxicity

Table 5 displays treatment-related toxicity. Chemotherapy-related grade 3 toxicity occurred in 12 patients (21.1%), with the most common being diarrhea, dehydration, and febrile neutropenia. Three patients (5.3%) could not tolerate their initial chemotherapy regimen and were switched to a new regimen. Clavien-Dindo grade 3b toxicity occurred in 2/38 patients (5.3%) who underwent surgical resection. One patient developed a gastroduodenal artery pseudoaneurysm roughly seven weeks after surgery, requiring coil embolization. The other patient developed a gastrocutaneous fistula approximately three weeks after surgery, requiring endoscopic guided suturing. Acute grade 1 radiation toxicity was observed in 26 patients (45.6%) and acute grade 2 radiation toxicity was observed in four patients (7.0%). The most common acute radiation toxicities were fatigue, nausea/vomiting, and constipation. Three patients (5%) experienced late grade 3 radiation toxicity. One patient developed gastric outlet obstruction 17 months after SBRT, with endoscopy showing delayed gastric emptying and redundant thick folds of gastrojejunosotomy wall. A gastrostomy tube was placed, and the patient was discharged home. Maximum dose to the stomach and jejunum was 13.8 Gy and 27.9 Gy, respectively. Another patient developed gastrointestinal bleeding from a duodenal ulcer roughly eight months after SBRT. This patient was successfully treated with endoscopic guided electrocoagulation therapy and epinephrine injection and discharged home in a stable condition. Maximum dose to the duodenum was 38.0 Gy. The final patient experienced gastrointestinal bleeding from both a splenic artery pseudoaneurysm and the pancreaticojejunostomy approximately 17 months after SBRT, requiring interventional radiology guided splenic artery coil embolization. Maximum dose to the jejunum was 35.5 Gy. There were no events of CTCAE grade > 4 or Clavien-Dindo grade > 4 toxicity.

Supplementary Table S4 shows toxicity rates of the study population (≥70 years) and the rest of the source population (<70 years). There were no differences in chemotherapy-induced, Clavien-Dindo 3b, and radiation-induced toxicity between the two groups.

## 4. Discussion

In this study, we demonstrate that in older patients who complete chemotherapy, aggressive local treatment with SBRT and surgery is well tolerated, with just three events (5.3%) of late grade 3 radiation toxicity and two events (5.3%) of Clavien-Dindo grade 3b toxicity in those who underwent resection. We also show that surgical resection is associated with improved clinical outcomes such as OS, DMFS and PFS, including an impressive median OS of 29.1 months.

Older patients with pancreatic cancer represent a challenging population given the aggressive nature of the disease and because many will not be candidates for multimodality therapy due to co-morbidities and poor performance status. As such, older patients (≥70 years) are underrepresented in clinical trials, accounting for 46% of the United States cancer population, but compromising only 20% of trial participants [7,8]. As such, the optimal management for these patients is unknown. Furthermore, many older patients do not receive standard of care treatment. Studies have shown that older patients are less likely to undergo surgical resection and receive adjuvant chemotherapy [19,20,21]. In addition, a study by Krzyzanwoska et al. of LAPC, demonstrated that only 44% of older patients received any form of treatment [13]. Therefore, additional studies are warranted to determine the most appropriate management for this group of patients.

Several studies have demonstrated the feasibility of SBRT in older patients and are shown in Table 6 [14,15,16,17,18]. Three studies, including the current study, included older patients who underwent surgery. While cross-study comparisons are limited and our cohort was likely more fit given that all patients completed chemotherapy, the fact that a large proportion (67%) underwent surgical resection is noteworthy and higher than other studies (34%, 10%) [16,18]. This likely led to the higher median OS in our patients (19.6 versus 14.0 and 10–13 months) [16,18]. Conversely, the median OS was < 10 months in the studies, which included only inoperable patients [14,15,17]. In the current report, patients who underwent resection had improved median OS (29.1 vs. 7.0 months, *p* = 0.005). Sutera et al. demonstrated similar results (28.3 vs. 11.4 months, *p* = 0.002) [18]. Interestingly, the disease extent was similar in our study (BRPC: 47.4%, LAPC: 52.6%) to that of Sutera et al. (resectable/BRPC: 46.2%, LAPC: 53.8%) and Zhu et al. (BRPC: 32.5%, LAPC: 67.5%). The difference in resection rate can likely be attributed to institution-specific surgical practice patterns. For example, at our institution, all BRPC/LAPC are taken for surgical exploration as long as they have stable disease, no medical contraindications, and if there is a reasonable pathway for resection. Additionally, our cohort was likely more fit given that we selected for patients who completed chemotherapy. Of the 19 patients who did not undergo resection in our cohort, only two had medical contraindications/advanced age. Of the 38 patients who underwent resection, 22 (57.9%) and 2 (5.3%) experienced Clavien-Dindo grades < 3a and 3b toxicity, respectively, which is consistent with rates from other series, which included patients of all ages [22,23]. These findings suggest that surgery can be well tolerated in older in patients who complete chemotherapy and should not be withheld solely based on age criteria, as resection may significantly improve outcomes in these patients.

Unfortunately, some older patients are unfit for surgical resection or aggressive systemic therapy. These patients may be at risk of uncontrolled tumor growth, which can cause significant local morbidity such as cholangitis, biliary obstruction, GI bleeding, and celiac plexopathy, which in turn, can drive both morbidity and mortality [24]. In fact, the median OS for untreated pancreatic cancer is just 2.5 months [25]. Therefore, minimally invasive treatments, such as SBRT, are important for this group of patients. Several studies have investigated the role of SBRT in inoperable older patients, with encouraging results [14,15,17]. Kim et al. reported on 26 patients with a median age of 86 years, who were treated with SBRT + chemotherapy [14]. They reported a median OS of 7.6 months and 1-year LPFS rate of 41.2%, with no late grade > 3 toxicity. Of note, there was a 70% rate of symptom relief after SBRT. Ryan et al. reported on 29 patients with inoperable disease treated with SBRT + chemotherapy [17]. They reported a median OS of 8.0 months and 1-year LPFS of 78%, with a symptom relief rate of 58%. There was only one episode of late grade 3 toxicity. In the current study, 19 patients underwent SBRT alone, with a median OS of 7.0 months and one-year LPFS of 72.0%. There were no late grade > 3 radiation toxicity events. Interestingly, the one-year LPFS rate reported by Kim et al. is significantly lower, despite utilizing a higher median biologically effective dose. One explanation is that a lower percentage (48%) of patients received chemotherapy when compared to the study by Ryan et al. (83%) and our study (100%), highlighting the role of systemic therapy in local control. Taken together, these findings demonstrate that SBRT for older patients with medically inoperable/unresectable disease is safe, feasible, and can provide adequate symptom relief.

One area of active investigation, which may be relevant to older patients, is dose escalation for inoperable pancreatic cancer. Retrospective data has shown that increased radiation dose can improve both OS and LPFS [26,27,28,29]. Studies, including the current one, demonstrate that SBRT to conventional doses is safe and feasible, but that outcomes can be suboptimal especially in older patients who cannot undergo surgical resection or receive systemic therapy [14,15,16,17,18]. Therefore, dose-escalated therapy may be of benefit in these patients. However, very little is known about the safety of increased radiation dose in older patients. Additionally, the prolonged treatment time associated with dose escalation must weighed against the limited life expectancy and potential detriment to quality of life in these patients. As such, further studies investigating the safety and efficacy of dose escalation in this older population is warranted.

There are some limitations of the current study, including its retrospective design. Patients received various chemotherapy regimens, which may have impacted clinical outcomes, although this was controlled for during statistical analysis. Our study population was also likely more fit when compared to others, because we only included patients who completed chemotherapy, limiting cross-study comparisons. Moreover, it also would have been useful to demonstrate underutilization of local therapies in elderly patients as compared to other age groups, certainly a worthwhile subject of future study, but this was not possible with our institutional data. Furthermore, some patients had follow-up locally at outside institutions, likely leading to underreporting of treatment-related toxicity. We were also unable to comment on symptom palliation as this information was not available for review. The strengths of this study include its relatively large sample size, homogenous treatment characteristics with regards to general treatment paradigm and SBRT dose/fractionation, and long follow-up time. Despite the study’s limitations, the findings add relevant information to the role of SBRT in the treatment of older patients.

## 5. Conclusions

In conclusion, we demonstrate that multimodality therapy in older patients with localized pancreatic cancer is safe and feasible and that surgical resection can improve clinical outcomes. As such, older patients who tolerate chemotherapy should not be excluded from aggressive local therapy with SBRT and surgery when possible. Finally, dose escalation for non-operable older patients may be appealing, but further studies investigating its safety are needed.

## Figures and Tables

**Table 1:** Patient, treatment, and disease characteristics.

Characteristics	N (%) or Median (Range)
No. of Patients	57
Age (years)	73.6 (70.1–84.1)
Sex	
Male	31 (54.4)
Female	26 (45.6)
ECOG	
0	17 (29.8)
1	38 (66.7)
2	2 (3.5)
ACE-27	
0	11 (19.3)
1	35 (61.4)
2	9 (15.8)
3	2 (3.5)
Histology	
Adenocarcinoma	55 (96.4)
Acinar cell	1 (1.8)
Undifferentiated carcinoma	1 (1.8)
Location of primary tumor	
Head	33 (57.9)
Other	24 (42.1)
Disease extent	
Borderline resectable	27 (47.4)
Locally advanced	30 (52.6)
Baseline CA 19-9 (U/mL)	233.3 (1.0–7358.4)
Baseline total bilirubin (U/m	0.55 (0.2–15.2)
Induction chemotherapy duration (months)	4 (2–18)
Induction chemotherapy	
mFFX	30 (52.5)
GnP	24 (42.1)
mFFX and capecitabine	1 (1.8)
Gemcitabine and capecitabine	1 (1.8)
Gemcitabine	1 (1.8)
SBRT dose and fractionation	
33 Gy in 5 fractions	53 (93.0)
30 Gy 5 fractions	2 (3.5)
36 Gy in 5 fractions	2 (3.5)
PTV (cm^3^)	108.8 (13.1–368.8)
Surgically Resected	38 (66.7)
Whipple	27 (71.1)
Distal	10 (26.3)
Total pancreatectomy	1 (2.6)
Post-SBRT/surgery chemotherapy	
Yes	19 (33.3)
No	38 (66.7)

Abbreviations: ECOG, Eastern Cooperative Oncology Group; CA 19-9, cancer antigen 19-9; mFFX, modified FOLFIRINOX; GnP, gemcitabine plus nab-paclitaxel; SBRT, stereotactic body radiation therapy; PTV, planning target volume.

**Table 2:** Practice patterns in older patients and rest of the source population.

	Older Patients (≥70 Years)	Rest of the Source Population (<70 Years)	
Variable	N (% or Range)	N (% or Range)	*p* Value
Total number	57	125	
Multi-agent chemotherapy	56 (98.2)	125 (100)	0.127
Chemotherapy regimen			
mFFX	31 (54.4)	111 (88.8)	**<0.001**
GnP	24 (42.1)	14 (12.2)	**<0.001**
Gemcitabine/capecitabine	1 (1.8)	0 (0)	0.127
Gemcitabine	1 (1.8)	0 (0)	0.127
Median chemotherapy duration (months)	4 (2–18)	4 (1–15)	0.233
Surgical Exploration	43 (75.4)	111 (88.8)	**0.025**
Resected	38 (66.7)	86 (68.8)	0.775
Post-SBRT/surgery chemotherapy	19 (33.3)	45 (36.0)	0.726

Abbreviations: mFFX, modified FOLFIRINOX; GnP, gemcitabine plus nab-paclitaxel. Bold text indicates *p*-value < 0.05.

**Table 3:** Univariate and multivariable analyses of overall survival.

	UVA			MVA		
	HR	95% CI	*p*	HR	95% CI	*p*
Age (years)	1.06	0.96–1.16	0.259			
Sex (male vs. female)	0.45	0.21–0.97	**0.042**	1.51	0.61–3.69	0.371
ECOG (0 vs. 1–2)	0.99	0.45–2.20	0.981			
ACE-27 (0–1 vs. 2–3)	0.35	0.16–0.74	**0.007**	0.82	0.28–2.39	0.722
Disease extent (BRPC vs. LAPC)	1.36	0.66–2.81	0.403			
Tumor location (head vs. other)	0.66	0.32–1.35	0.256			
Induction CT duration (>4 vs. ≤4 months)	0.63	0.29–1.34	0.229			
Induction CT (mFFX vs. GnP)	1.29	0.60–2.76	0.516			
Resected (yes vs. no)	0.20	0.092–0.42	**<0.001**	0.30	0.12–0.91	0.031
Baseline CA 19-9 (U/mL)	0.99	0.99–1.00	0.883			
Pre-SBRT CA 19-9 (U/mL)	1.00	0.99–1.00	0.633			
Baseline Total Bilirubin (mg/dL)	1.00	0.88–1.10	0.989			
Post-SBRT/surgery chemotherapy (yes vs. no)	0.26	0.11–0.64	**0.004**	0.41	0.15–1.14	0.087

Abbreviations: ECOG, Eastern Cooperative Oncology Group; ACE-27, adult comorbidity evaluation-27; BRPC, borderline resectable pancreatic cancer; LAPC, locally advanced pancreatic cancer; mFFX, modified FOLFIRINOX; GnP, gemcitabine plus nab-paclitaxel; CA 19-9, cancer antigen 19-9; SBRT, stereotactic body radiation therapy. Bold text indicates *p*-value < 0.05.

**Table 4:** Univariate and multivariable analyses of progression-free survival.

	UVA			MVA		
	HR	95% CI	*p*	HR	95% CI	*p*
Age (years)	1.09	1.01–1.18	**0.034**	1.02	0.93–1.12	0.791
Sex (male vs. female)	1.89	1.04–3.46	**0.038**	1.49	0.74–3.00	0.261
ECOG (0 vs. 1–2)	0.65	0.33–1.25	0.196			
ACE-27 (0–1 vs. 2–3)	0.33	0.16–0.66	**0.002**	0.56	0.23–1.34	0.193
Disease extent (BRPC vs. LAPC)	1.24	0.69–2.23	0.476			
Tumor location (head vs. other)	0.94	0.52–1.69	0.827			
Induction CT duration (>4 vs. ≤4 months)	0.95	0.52–1.72	0.861			
Induction CT (mFFX vs. GnP)	0.99	0.54–1.82	0.980			
Resected (yes vs. no)	0.25	0.13–0.47	**<0.001**	0.40	0.17–0.93	0.034
Baseline CA 19-9 (U/mL)	1.00	0.99–1.00	0.387			
Pre-SBRT CA 19-9 (U/mL)	1.00	1.00–1.00	0.405			
Baseline Total Bilirubin (mg/dL)	1.02	0.91–1.10	0.729			
Post-SBRT/surgery chemotherapy (yes vs. no)	0.43	0.22–0.82	**0.011**	0.58	0.28–1.19	0.140

Abbreviations: ECOG, Eastern Cooperative Oncology Group; ACE-27, adult comorbidity evaluation-27; BRPC, borderline resectable pancreatic cancer; LAPC, locally advanced pancreatic cancer; mFFX, modified FOLFIRINOX; GnP, gemcitabine plus nab-paclitaxel; CA 19-9, cancer antigen 19-9; SBRT, stereotactic body radiation therapy. Bold text indicates *p*-value < 0.05.

**Table 5:** Treatment related toxicity.

	N (%)	Description
Chemotherapy toxicity		
Grade 3	12 (21.1%)	Diarrhea (4), dehydration (3), febrile neutropenia (3), pneumonitis (1), neuropathy (1), CHF exacerbation (1), fatigue (1), nausea (1)
Surgical toxicity (<90 days)		
Clavien-Dindo grade ≤ 3a	22 (57.9%)	
Clavien-Dindo grade 3b	2 (5.3%)	Gastroduodenal artery pseudoaneurysm, gastrocutaneous fistula
Radiation toxicity		
Acute		
Grade 1	26 (45.6%)	Fatigue (16), nausea/vomiting (15), constipation (7), anorexia (4), pain (4)
Grade 2	4 (7.0%)	Pain (2), fatigue (1), anorexia (1)
Grade 3	0 (0%)	
Grade 4	0 (0%)	
Late		
Grade 1	0 (0%)	
Grade 2	0 (0%)	
Grade 3	3 (5.3%)	Upper gastrointestinal bleeding (2), gastric outlet obstruction (1)
Grade 4	0 (0%)	

Abbreviations: CHF, congestive heart failure.

**Table 6:** Literature on SBRT for older patients.

Reference	Relevant Patients	Median Age (Years)	Median SBRT Dose/Fractions	Surgical Resection	Acute Radiation ≥ G3 Toxicity	Late Radiation ≥ G3 Toxicity	Median Survival after SBRT (Months)
Kim et al. (2013) [14]	24	86	24 Gy/1 fraction	No	1 (4%)	0 (0%)	7.6
Yechieli et al. (2017) [15]	20	83	35 Gy/5 fractions	No	1 (10%)	2 (10%)	6.4
Zhu et al. (2017) [16]	323	73	30–46.8 Gy/5–8 fractions	Yes (10%)	2 (0.5%)	0 (0%)	10.0–13.0
Ryan et al. (2017) [17]	29	74	28 Gy/5 fractions	No	2 (7%)	1 (3%)	8.0
Sutera et al. (2018) [18]	145	79	36 Gy/3 fractions	Yes (34%)	1 (1%)	3 (2%)	14.0
Current Study	57	74	33 Gy/5 fractions	Yes (67%)	0 (0%)	3 (5%)	19.6

## Data Availability

Data will be shared upon request to the corresponding author and after approval by the Institutional Review Board of Johns Hopkins University.

## Supplementary Materials

The following supporting information can be downloaded at: https://www.mdpi.com/article/10.3390/curroncol29010028/s1, Figure S1: Kaplan Meier curves of (A) local progression-free survival, (B) local progression-free survival based on resection status, (C) distant metastasis-free survival, and (D) distant metastasis-free survival based on resection status. Table S1: Practice patterns and toxicity in older patients based on age cutoffs. Table S2: Univariate and multivariable analyses of local progression-free survival. Table S3: Univariate and multivariable analyses of distant metastasis-free survival. Table S4: Toxicity in older patients and rest of the source population.

## References

[1] Siegel R.L., Miller K.D., Fuchs H.E., Jemal A. (2021). Cancer Statistics, 2021. CA Cancer J. Clin..

[2] Rahib L., Wehner M.R., Matrisian L.M., Nead K.T. (2021). Estimated Projection of US Cancer Incidence and Death to 2040. JAMA Netw Open.

[3] National Comprehensive Cancer Network Pancreatic Adenocarcinoma. Version 2.2021. https://www.nccn.org/professionals/physician_gls/pdf/pancreatic_blocks.pdf.

[4] Rawla P., Sunkara T., Gaduputi V. (2019). Epidemiology of Pancreatic Cancer: Global Trends, Etiology and Risk Factors. World J. Oncol..

[5] National Cancer Institute (2008). SEER Cancer Statistics Review, 1975–2018.

[6] Pratt W.B., Gangavati A., Agarwal K., Schreiber R., Lipsitz L.A., Callery M.P., Vollmer C.M. (2009). Establishing Standards of Quality for Elderly Patients Undergoing Pancreatic Resection. Arch. Surg..

[7] Talarico L., Chen G., Pazdur R. (2004). Enrollment of Elderly Patients in Clinical Trials for Cancer Drug Registration: A 7-Year Experience by the US Food and Drug Administration. J. Clin. Oncol..

[8] Sedrak M.S., Freedman R.A., Cohen H.J., Muss H.B., Jatoi A., Klepin H.D., Wildes T.M., Le-Rademacher J.G., Kimmick G.G., Tew W.P. (2021). Older Adult Participation in Cancer Clinical Trials: A Systematic Review of Barriers and Interventions. CA Cancer J. Clin..

[9] Higuera O., Ghanem I., Nasimi R., Prieto I., Koren L., Feliu J. (2016). Management of Pancreatic Cancer in the Elderly. World J. Gastroenterol..

[10] Dindo D., Demartines N., Clavien P.-A. (2004). Classification of Surgical Complications: A New Proposal with Evaluation in a Cohort of 6336 Patients and Results of a Survey. Ann. Surg..

[11] Bouchardy C., Rapiti E., Blagojevic S., Vlastos A.-T., Vlastos G. (2007). Older Female Cancer Patients: Importance, Causes, and Consequences of Undertreatment. J. Clin. Oncol..

[12] Quaglia A., Tavilla A., Shack L., Brenner H., Janssen-Heijnen M., Allemani C., Colonna M., Grande E., Grosclaude P., Vercelli M. (2009). The Cancer Survival Gap between Elderly and Middle-Aged Patients in Europe Is Widening. Eur. J. Cancer.

[13] Krzyzanowska M.K., Weeks J.C., Earle C.C. (2003). Treatment of Locally Advanced Pancreatic Cancer in the Real World: Population-Based Practices and Effectiveness. J. Clin. Oncol..

[14] Kim C.H., Ling D.C., Wegner R.E., Flickinger J.C., Heron D.E., Zeh H., Moser A.J., Burton S.A. (2013). Stereotactic Body Radiotherapy in the Treatment of Pancreatic Adenocarcinoma in Elderly Patients. Radiat. Oncol..

[15] Yechieli R.L., Robbins J.R., Mahan M., Siddiqui F., Ajlouni M. (2017). Stereotactic Body Radiotherapy for Elderly Patients with Medically Inoperable Pancreatic Cancer. Am. J. Clin. Oncol..

[16] Zhu X., Li F., Ju X., Cao F., Cao Y., Fang F., Qing S., Shen Y., Jia Z., Zhang H. (2017). Prognostic Role of Stereotactic Body Radiation Therapy for Elderly Patients with Advanced and Medically Inoperable Pancreatic Cancer. Cancer Med..

[17] Ryan J.F., Rosati L.M., Groot V.P., Le D.T., Zheng L., Laheru D.A., Shin E.J., Jackson J., Moore J., Narang A.K. (2018). Stereotactic Body Radiation Therapy for Palliative Management of Pancreatic Adenocarcinoma in Elderly and Medically Inoperable Patients. Oncotarget.

[18] Sutera P.A., Bernard M.E., Wang H., Heron D.E. (2018). Prognostic Factors for Elderly Patients Treated With Stereotactic Body Radiation Therapy for Pancreatic Adenocarcinoma. Front Oncol..

[19] Nagrial A.M., Chang D.K., Nguyen N.Q., Johns A.L., Chantrill L.A., Humphris J.L., Chin V.T., Samra J.S., Gill A.J., Pajic M. (2014). Adjuvant Chemotherapy in Elderly Patients with Pancreatic Cancer. Br. J. Cancer.

[20] Sohn T.A., Yeo C.J., Cameron J.L., Lillemoe K.D., Talamini M.A., Hruban R.H., Sauter P.K., Coleman J., Ord S.E., Grochow L.B. (1998). Should Pancreaticoduodenectomy Be Performed in Octogenarians?. J. Gastrointest. Surg..

[21] Meguid R.A., Ahuja N., Chang D.C. (2008). What Constitutes a “High-Volume” Hospital for Pancreatic Resection?. J. Am. Coll. Surg..

[22] Blair A.B., Rosati L.M., Rezaee N., Gemenetzis G., Zheng L., Hruban R.H., Cameron J.L., Weiss M.J., Wolfgang C.L., Herman J.M. (2018). Postoperative Complications after Resection of Borderline Resectable and Locally Advanced Pancreatic Cancer: The Impact of Neoadjuvant Chemotherapy with Conventional Radiation or Stereotactic Body Radiation Therapy. Surgery.

[23] Gemenetzis G., Groot V.P., Blair A.B., Laheru D.A., Zheng L., Narang A.K., Fishman E.K., Hruban R.H., Yu J., Burkhart R.A. (2019). Survival in Locally Advanced Pancreatic Cancer After Neoadjuvant Therapy and Surgical Resection. Ann. Surg..

[24] Cardillo N., Seible D.M., Fero K.E., Bruggeman A.R., Sarkar R.R., Azuara A., Simpson D.R., Murphy J.D. (2018). Clinical Impact of Local Progression in Pancreatic Cancer. J. Natl. Compr. Cancer Netw..

[25] Glimelius B., Hoffman K., Sjödén P.O., Jacobsson G., Sellström H., Enander L.K., Linné T., Svensson C. (1996). Chemotherapy Improves Survival and Quality of Life in Advanced Pancreatic and Biliary Cancer. Ann. Oncol..

[26] Reyngold M., O’Reilly E.M., Varghese A.M., Fiasconaro M., Zinovoy M., Romesser P.B., Wu A., Hajj C., Cuaron J.J., Tuli R. (2021). Association of Ablative Radiation Therapy With Survival Among Patients With Inoperable Pancreatic Cancer. JAMA Oncol..

[27] Krishnan S., Chadha A.S., Suh Y., Chen H.-C., Rao A., Das P., Minsky B.D., Mahmood U., Delclos M.E., Sawakuchi G.O. (2016). Focal Radiation Therapy Dose Escalation Improves Overall Survival in Locally Advanced Pancreatic Cancer Patients Receiving Induction Chemotherapy and Consolidative Chemoradiation. Int. J. Radiat. Oncol. Biol. Phys..

[28] Golden D.W., Novak C.J., Minsky B.D., Liauw S.L. (2012). Radiation Dose ≥54 Gy and CA 19-9 Response Are Associated with Improved Survival for Unresectable, Non-Metastatic Pancreatic Cancer Treated with Chemoradiation. Radiat. Oncol..

[29] Liauw S.L., Ni L., Wu T., Arif F., Cloutier D., Posner M.C., Kozloff M., Kindler H.L. (2020). A Prospective Trial of Stereotactic Body Radiation Therapy for Unresectable Pancreatic Cancer Testing Ablative Doses. J. Gastrointest. Oncol..

