# Supplementary material for: Upfront Chemotherapy Followed by Stereotactic Body Radiation Therapy with or without Surgery in Older Patients with Localized Pancreatic Cancer: A Single Institution Experience and Review of the Literature
Source: Curr Oncol. 2022 Jan 11;29(1):308–20. doi: 10.3390/curroncol29010028 (PMC8774377; doi:10.3390/curroncol29010028)
Supplement: Supplementary file 1 [file curroncol-29-00028-s001.zip › curroncol-1499117-SI.pdf]

Supplementary Materials:

**Supplementary Table 1.** Practice patterns and toxicity in older patients based on age cutoffs				
	**70-75**	**75-80**	**≥80**	**P value**
**Variable**	N (% or range)	N (% or range)	N (% or range)	
Total number	37	14	6	
Median age	72.4 (70.1-74.9)	77.7 (76.0-79.7)	80.9 (80.4-84.1)	
**Practice patterns**				
Multi-agent chemotherapy	37 (100)	13 (92.9)	6 (100)	0.239
Chemotherapy regimen				
mFFX	25 (67.6)	6 (42.9)	0 (0)	**0.002**
GnP	12 (32.4)	6 (42.9)	6 (100)	**0.003**
Gemcitabine/Capecitabine	0 (0)	1 (7.1)	0 (0)	0.239
Gemcitabine	0 (0)	1 (7.1)	0 (0)	0.239
Median chemotherapy duration (months)	4 (2-18)	4 (2-6)	4 (2-5)	0.279
Surgical Exploration	31 (83.8)	9 (64.3)	3 (50)	0.123
Resected	28 (75.7)	8 (57.1)	2 (33.3)	0.093
Post-SBRT/surgery chemotherapy	16 (43.2)	2 (14.3)	1 (16.7)	0.080
**Toxicity**				
Grade 3 chemotherapy toxicity	8 (21.6)	3 (21.4)	1 (16.7)	0.960
Clavien-Dindo 3b toxicity	0 (0)	1 (7.1)	1 (16.7)	0.095
Grade 3 radiation toxicity	1 (2.7)	2 (14.3)	0 (0)	0.243

Abbreviations: mFFX, modified FOLFIRINOX; GnP, gemcitabine plus nab-paclitaxel. Bold text indicates P-value < 0.05

**Supplementary Table 2.** Univariate and multivariable analyses of local progression-free survival						
	UVA			MVA		
	HR	95% CI	P	HR	95% CI	P
Age (years)	1.11	0.99-1.23	0.083			
Sex (male vs female)	1.59	0.67-3.79	0.296			
ECOG (0 vs 1-2)	1.35	0.57-3.22	0.492			
ACE-27 (0-1 vs 2-3)	0.75	0.22-2.62	0.657			
Disease extent (BRPC vs LAPC)	2.31	0.93-5.76	0.062			
Tumor location (head vs other)	1.15	0.48-2.73	0.750			
Induction CT duration (> 4 vs ≤ 4 months)	0.99	0.42-2.37	0.999			
Induction CT (mFFX vs GnP)	1.48	0.58-3.77	0.412			
Resected (yes vs no)	0.47	0.17-1.24	0.127			
Baseline CA 19-9 (U/ml)	1.00	0.99-1.00	0.071			
Pre-SBRT CA 19-9 (U/ml)	1.00	1.00-1.00	**0.037**			

Baseline Total Bilirubin (mg/dl)	1.08	0.95-1.18	0.216			
Post-SBRT/surgery chemotherapy (yes vs no)	0.43	0.16-1.11	0.081			

Abbreviations: ECOG, Eastern Cooperative Oncology Group: ACE-27, adult comorbidity evaluation-27; BRPC, borderline resectable pancreatic cancer; LAPC, locally advanced pancreatic cancer; mFFX, modified FOLFIRINOX; GnP, gemcitabine plus nab-paclitaxel; CA 19-9, cancer antigen 19-9; SBRT, stereotactic body radiation therapy. Bold text indicates P-value < 0.05

Supplementary Table 3. Univariate and multivariable analyses of distant metastasis-free survival

	UVA			MVA		
	HR	95% CI	P	HR	95% CI	P
Age (years)	1.05	0.96-1.14	0.275			
Sex (male vs female)	1.50	0.79-2.87	0.217			
ECOG (0 vs 1-2)	0.47	0.22-1.01	0.052			
ACE-27 (0-1 vs 2-3)	0.34	0.16-0.72	**0.005**	0.46	0.20-1.03	0.060
Disease extent (BRPC vs LAPC)	1.40	0.74-2.66	0.301			
Tumor location (head vs other)	0.97	0.51-1.87	0.929			
Induction CT duration (> 4 vs ≤ 4 months)	0.86	0.45-1.65	0.654			
Induction CT (mFFX vs GnP)	1.18	0.60-2.31	0.628			
Resected (yes vs no)	0.22	0.11-0.46	**<0.001**	0.33	0.14-0.77	**0.010**
Baseline CA 19-9 (U/ml)	0.99	0.99-1.00	0.894			
Pre-SBRT CA 19-9 (U/ml)	1.00	0.99-1.00	0.674			
Baseline Total Bilirubin (mg/dl)	0.99	0.87-1.09	0.909			
Post-SBRT/surgery chemotherapy (yes vs no)	0.47	0.23-0.94	**0.032**	0.61	0.28-1.32	0.207

Abbreviations: ECOG, Eastern Cooperative Oncology Group; ACE-27, adult comorbidity evaluation-27; BRPC, borderline resectable pancreatic cancer; LAPC, locally advanced pancreatic cancer; mFFX, modified FOLFIRINOX; GnP, gemcitabine plus nab-paclitaxel; CA 19-9, cancer antigen 19-9; SBRT, stereotactic body radiation therapy. Bold text indicates P-value < 0.05

Supplementary Table 4. Toxicity in older patients and rest of the source population

	**Older patients (≥ 70 years)**	**Rest of the source population (< 70 years)**	**P value**
	N (%)	N (%)	
**Total Number**	57	125	
**Toxicity**			
Grade 3 chemotherapy toxicity	12 (21.1)	17 (13.6)	0.211
Clavien-Dindo 3b toxicity	2 (5.3)	3 (3.5)	0.651
Grade 3 radiation toxicity	3 (5.3)	4 (3.2)	0.513

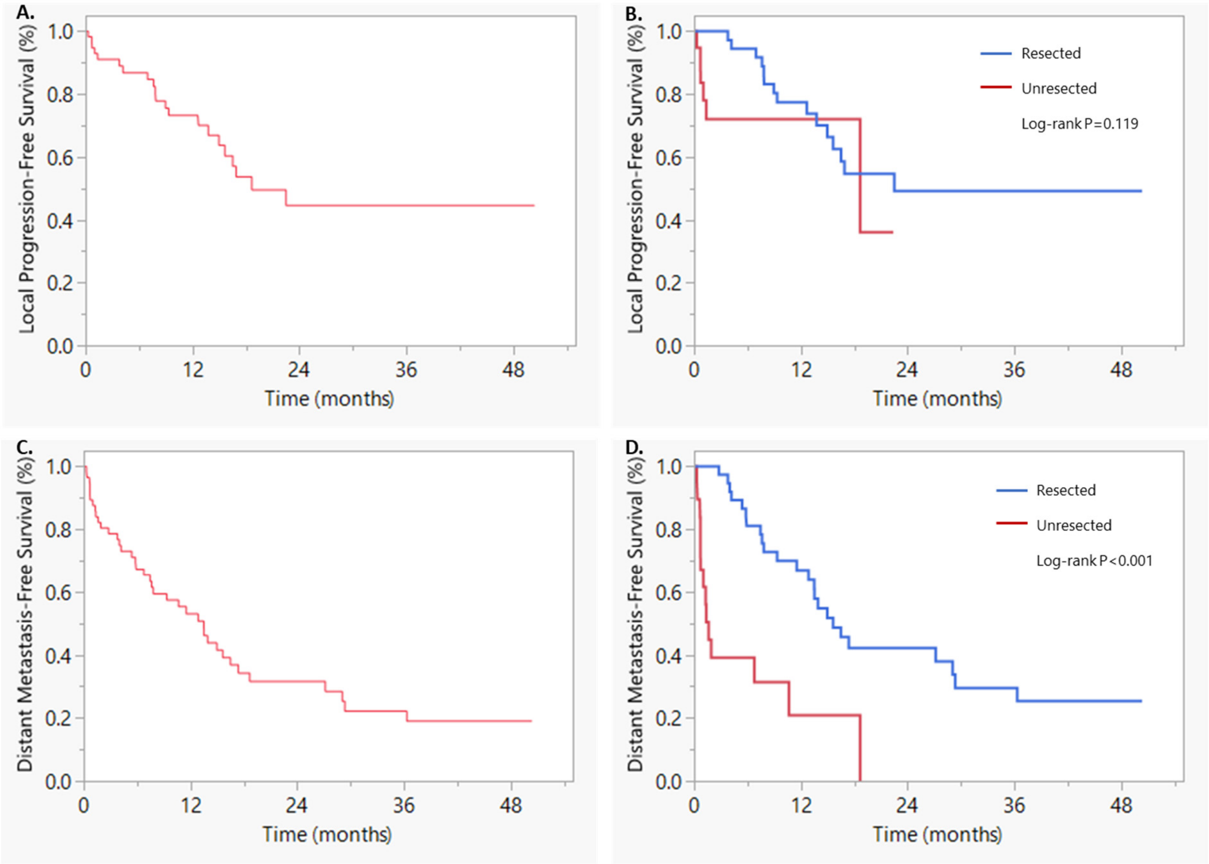

Supplementary Figure 1. Kaplan Meier curves of A) local progression-free survival, B) local progression-free survival based on resection status, C) distant metastasis-free survival, and D) distant metastasis-free survival based on resection status.
